# How do General Practitioners experience providing care for their psychotic patients?

**DOI:** 10.1186/1471-2296-8-37

**Published:** 2007-06-28

**Authors:** Marian JT Oud, Jan Schuling, Cees J Slooff, Betty Meyboom-de Jong

**Affiliations:** 1 Department of General Practice, University Medical Center Groningen, Groningen, The Netherlands; 2 Department of Psychiatry, University Medical Center Groningen, Groningen, The Netherlands; 3 Mental Health Center Drenthe, Assen, The Netherlands

## Abstract

**Background:**

In primary care, GPs usually provide care for patients with chronic diseases according to professional guidelines. However, such guidelines are not available in the Netherlands for patients with recurring psychoses. It seems that the specific difficulties that GPs experience in providing care for these patients hinder the development and implementation of such guidelines. This study aims to explore the chances and problems GPs meet when providing care for patients susceptible for recurring psychoses, including schizophrenia and related disorders, bipolar disorder, and psychotic depression.

**Methods:**

A qualitative study of focus group discussions with practising GPs in both town and rural areas. Transcripts from three focus groups with 19 GPs were analysed with the computer program 'Kwalitan'. Theoretical saturation was achieved after these three groups.

**Results:**

Analysis showed that eight categories of factors influenced the GPs' care for psychotic patients: patient presentation (acute vs. chronic phase), emotional impact, expertise, professional attitude, patient related factors, patient's family, practice organization, and collaboration with psychiatric specialists.

**Conclusion:**

Current primary care for psychotic patients depends very much on personal characteristics of the GP and the quality of local collaboration with the Mental Health Service. A quantitative study among GPs using a questionnaire based on the eight categories mentioned above would determine the extent of the problems and limitations experienced with this type of care. From the results of this quantitative study, new realistic guidelines could be developed to improve the quality of care for psychotic patients.

## Background

Chronic, relapsing psychoses are psychiatric diseases which have serious implications for the quality of life of both patients and those around them. Usually the general practitioner (GP) is the first contact when disturbing symptoms occur that are severe enough to cause alarm within the family. When the GP recognizes the psychosis and the patient allows himself to be referred to a psychiatrist, the GP becomes a concerned observer. Information is received from the family, the patient, and the specialist.

The GP is the first to be consulted if a patient has physical complaints. However, patients with chronic psychoses are less capable than other patients of interpreting physical signs, as well as solving their problems and caring for themselves, which leads to an increased responsibility on the part of health care workers. For example, the co-morbid conditions of schizophrenia include anxiety, drugs and alcohol abuse, weight gain, diabetes mellitus, cardiovascular diseases, and general consequences of an unhealthy lifestyle [1,2]. In short, this is a group of patients who are chronically ill and for whom the GP is a convenient provider of continuing care with easy access. As with other chronic conditions, guidelines for the primary care of patients with schizophrenia would improve the quality of care [3,4]. The implementation of a protocol outlining such guidelines requires that the interventions are realistic and that the GP is willing to apply them. Or as Paul Rowlands states: implementation is likely to be best achieved through a sense of ownership of the guidelines and a process that borrows from their spirit, which emphasises collaboration, building on the strengths and good practice already present [5,6]. Research has shown, however, that GPs have little interest in psychotic conditions and patients with schizophrenia are less likely to receive physical health checks in primary care [7,8].

A qualitative study was planned which explored what factors affect the willingness of GPs to provide care for patients susceptible to recurring psychoses, including schizophrenia and related disorders, bipolar disorder, and psychotic depression. These results should be taken into account when developing guidelines. The research question was formulated as follows: what variables influence the GP when he or she is caring for patients with psychoses in the acute and chronic phase?

## Methods

As this study concerns a relatively uninvestigated area, a qualitative design with focus groups was chosen as research method. It was necessary to explore several aspects of the GPs' care (table 1), and pay particular attention to the problems experienced by GPs [9]. To obtain the possibly varying opinions, the participating GPs should differ in age, gender, and type of practice [10]. The participants were selected by purposeful sampling. A majority of experienced GPs was preferred so as to ensure that our group of respondents had sufficient experience in treating patients with psychoses. Practising GPs were invited by one of the investigators (MO) to participate in the study. All focus group meetings were led by a single moderator: a psychologist and senior staff member of the Department of Vocational Training of General Practice UMCG. Two investigators (MO and JS) working independently, categorized the fragments of text and divided them into themes. The discussions were recorded digitally and later transcribed. Any discrepancies were discussed until a consensus was reached. The analysis was conducted to the rules of qualitative research, based on the elements of grounded theory [11,12]. The software program Kwalitan was used to assign codes to each of the textual segments and to structure them in a decision tree format.

**Table 1:** Questions discussed in the focus groups

What experiences do you have with psychotic patients, and how do you feel about providing care for these patients?
How do you cope with a first and a recurring psychosis?
Pharmacotherapy, do you feel confident with it?
Have you developed a specific policy in counselling/supporting chronic psychotic patients? Who takes the initiative in making contact?
What kind of support and collaboration with the members of the family is there?
Do you feel competent in providing care for psychotic patients? If not, what sort of training do you need?
How do you feel about collaborating with the Mental Health Service?
How much satisfaction do you gain from providing care for psychotic patients?

## Results

A total of fifty GPs was approached, of whom 19 agreed to participate (table 2). GPs who declined to participate found the time investment (90 minutes excluding travel time) too great.

**Table 2:** The participants

Focus group 1			
Nr.	Sexe	Years of experience	Location
1	Female	20–25	Suburban area
2	Male	15–20	Suburban area
3	Male	5–10	City
4	Female	5–10	Suburban area
5	Male	20–25	City

Focus group 2			

6	Male	20–25	City
7	Female	20–25	City
8	Female	5–10	City
9	Male	10–15	City
10	Female	10–15	Rural area
11	Male	> 25	Rural area
12	Male	20–25	City
13	Male	20–25	City

Focus group 3			

14	Male	20–25	City
15	Female	5–10	City
16	Female	15–20	City
17	Female	20–25	City
18	Male	5–10	City
19	Female	0–5	Locum

Three focus groups were organized: two in Groningen and one in Amsterdam. Since the third focus group generated no new aspects and saturation was reached, no further groups were organized.

Nine female and ten male GPs participated and all except one had considerable professional experience, from 7 to more than 25 years. One GP was a locum. Thirteen GPs had their practice in the city, three were practising in suburban areas, and two had rural practices.

The analysis of the data resulted in eight categories being identified: patient presentation, the emotional impact, the expertise, the professional attitude, patient-related factors, the patient's family, the organization of the practice, and the collaboration with secondary health care sources.

### Patient presentation

When I have been asked to intervene because the patient's behaviour makes the situation unbearable, I usually find the patient in an embarrassing condition in which my abilities as a GP are very restricted. (PG2)

Psychosis presents itself in many forms: from dramatic crisis situations to calm, clear situations that include patients with chronic schizophrenia who reliably take their antipsychotic medication. Also, GPs encounter addicted patients who display compulsive or threatening behaviour, patients who are vulnerable to bouts of psychotic depression, and patients who are excited and manic. Some GPs also mentioned elderly patients, who hallucinate or are paranoid due to an organic brain syndrome and who require a lot of GP support. The GPs made an important distinction between the acute and the chronic phase of psychosis. Although acute psychotic crises rarely present themselves in the general practice setting, when they do occur, they have a large impact on the GP and on the organization of the practice.

### The emotional impact on the GP

The experience which left the biggest impression on me involved a father going through a psychotic crisis. He was threatening to take this tiny baby away with him. The mother tried to grab the child back, in the living room, and I uh... I contacted the Mental Health Service and waited for help. (GP7)

It is the GP's responsibility to do the initial examination during a psychotic episode. The GP's role in the actual treatment is limited, however. Usually, family members are the ones who seek help from the GP. The GP then responds by assessing the patient's mental state with an interview or information obtained from the family and his own observations upon arrival at the scene. In a crisis, GPs try to take the lead and restore order, but since they have limited experience with psychotic crises, they often feel powerless and depend on a quick response by the Mental Health Service team. The case reports discussed in the focus groups evoked a great amount of recognition of the problems and clearly demonstrated the need for emotional support. Most GPs feel alone, particularly when facing an acute crisis.

What the GPs experience and feel during a psychotic crisis involving a specific patient may have a lasting effect on the GPs' future attitudes towards that patient when caring for him or her in the chronic phase of the illness.

And when the patient doesn't want to be referred, you can only stand back and watch; and if he also refuses medication, you can only hope that things stay calm... (GP17)

Besides the feelings of powerlessness, anxiety also plays a role. GPs worry that patients with disturbed aggression management will become violent. They fear for their own personal safety, for disturbances in the GP surgery, and for frightening other patients. One GP mentioned that his anxiety was a result of feeling pulled into the psychotic thoughts of the patient.

Of course I get scared.... with menacing people. A man once came to my practice with very strange complaints. He was very intimidating without actually becoming violent, yes, threatening and incomprehensible behaviour... (GP6)

Three GPs find great satisfaction in caring for patients with schizophrenia, particularly during peaceful periods when the patient is stable. This satisfaction also depends on how knowledgeable the patient is about his disease, how easy it is to talk to him.

Look, if he has something to do during the day, and he receives reasonable care, and the situation doesn't get out of hand, then I enjoy it too: when a certain amount of continuity is achieved, then I enjoy my job. (GP3)

### The GP's expertise

GPs find themselves able to recognize a psychosis. However, with a new psychosis it can take some time before the clinical picture is correctly interpreted. GPs do not apply specific DSM categories, since they regard diagnosing according to these categories the psychiatrist's responsibility.

All GPs in this study refer young patients with a psychosis to a psychiatrist, while at the same time, they are willing to manage themselves older patients whose outlook is limited, and who will benefit from supportive and palliative care.

Most GPs expressed the view that they have a limited knowledge of pharmacotherapy. Starting a patient with schizophrenia on antipsychotic medication is considered to be the psychiatrist's responsibility. The GP's practice is usually involved in restarting medication or continuing prescription. For example, if experience with a previous antipsychotic drug has been positive, the GP will prescribe the same medication. In case of a newly diagnosed psychotic patient, six out of the 19 GPs will start the patient on antipsychotic medication themselves, whereas other GPs are inclined to refer the patient to specialized care immediately. GPs who initiate pharmacotherapy themselves hope to stabilize the patient, so that he or she is open to communication and is able to agree to the referral to a specialist.

You have built a relationship with the patient, and if you can make use of that, then you should; you shouldn't refrain from treatment because then you run the risk of delay. You have to minimize delay as much as possible, so just start... (GP3)

Three of the GPs expressed the desire to learn strategies for communicating with psychotic patients: 'how do I connect with these people, how do I tell someone that he or she is not rational, and what should my approach be?'

*Communication problems are inherent to schizophrenia, this is an important aspect of the disease: how do I communicate with these people...?  If they are really confused, then you have to take a different approach........ interview them differently from one who is coherent. (GP7)*

### The GP's professional approach

General practice offers people an easy and non-stigmatising access to health care.

It is a matter of sensitivity, and being there for them. I think this is the most important thing that you can be for the chronic psychiatric patient: that they can come to you and that they know that you know everything that has happened, and that they don't have to explain everything....... that they can trust you. (GP9)

The GP knows the context and background of most of his patients and maintains the patient's medical file. When problems occur, the GP is the first to respond, and he often functions as the final safety net. However, GPs are accustomed to working independently, and they generally do not have the opportunity to discuss more difficult patients with a team.

In the chronic phase, the practice assistant takes care of repeat prescriptions and the GP only becomes involved by request. Three GPs mentioned that they make use of repeat prescription requests to invite the patient for monitoring.

'Well no, says my assistant, I can't give you so many pills. It's time for you to see the doctor'. That is just the way we do it, it is an informal policy. Yes, just to see how things are going... (GP16)

To build an effective doctor-patient relationship, the GP chooses to maintain a neutral but interested attitude. Most GPs do not actively follow patients with a chronic psychosis. Only a few offer the occasional follow-up appointment or ask the patient to come in when he or she forgets to pick up a new prescription.

### Patient related factors

The patient's age and his prognosis play an important role in the care provided by GPs. Young people are always referred to the psychiatric specialist. People with a stable social context are referred less often than those without one. GPs with an affinity for chronic psychiatry feel ambivalent about their duties towards schizophrenic patients who refuse treatment and who have been rejected by society.

Patients who are not in treatment and whom I worry about......... and at whom nobody else bats an eyelid, what should I do with them............. (GP14)

The GPs do not know what to do with patients who refuse every form of treatment. When the patient has an aggressive attitude, the doctor may decide that he is unable to handle the patient. There was a consensus among participants on the need for close monitoring of aggressive psychotic patients by the Mental Health Service.

### The patient's family

As GP you are also the family doctor and I think that is a strong trump because you are very much involved with the family. You give them support and advice on how to deal which leads to an indirect coaching of the patient. (GP1)

The GP is a family physician, and all GPs consider it their responsibility to provide support and care for all the members of the patient's family. The family of a schizophrenic patient experiences stress and visits their GP more often [13,14]. Several GPs commented that, with certain patients, they invested considerable energy in caring for the patient's family during the acute psychotic phase. This concerned situations involving the family where the GP was trying to restore the balance in the family. Furthermore, contact with the family is important during the initial interviews, making appointments to discuss therapy compliance, and explaining the early recognition of symptoms indicating relapse.

### The organization of the general practice

The GP operates without protection and bears full responsibility. House calls and office appointments are planned in the short term, and the days pass according to a fixed schedule. A crisis, however, happens without warning and is very time consuming. This upsets the practice routine, necessitating overtime hours.

A psychotic crisis is very time consuming and disturbs surgery hours. You all know what a full Monday schedule brings about.... (GP6)

To be able to manage a psychotic crisis at home, GPs find it essential that a doctor-family relationship exists which allows reasonably reliable agreements to be made. It is equally important that the situation is surveyable and that the Mental Health Service is available to assist. In hindsight, two GPs judged the responsibility of dealing with a psychotic crisis and the time required for it to be considerable. A social-psychiatric nurse is available to some of the GPs in their practices for counselling patients and to act as a consultant for the GP, but this nurse usually has no experience of caring for patients with psychotic disorders.

During the evening and at night, emergencies are covered by GPs working from a centralised on-call centre. These on-call GPs can ask for assistance of a social-psychiatric nurse from the Mental Health Service if necessary.

### Collaborating with psychiatric specialists

This topic led to heated discussions, in which feelings of satisfaction, frustration, and powerlessness were expressed. Positive experiences were recounted involving nurses from the Mental Health Service who were willing and ready to assist and cooperate with the GP in solving problems. The GPs highly valued consultations with specialists and achieving shared care. Frustration following an inaccurate assessment by mental health care services is long felt by the GP who calls on acute psychiatric services, as well as feelings of being misunderstood.

Sometimes I feel extremely powerless when it comes to the crisis service.... then I think... I have been a GP for more than 15 years.., I know what's wrong with this patient! And nothing is done. And then I'm the one who finds her dead on the sidewalk the next day! (GP 16)

GPs do not feel their work is truly valued by the mental health care workers. They mentioned examples in which they felt excluded from the care of a patient. However, one GP was quite satisfied with an emergency plan developed by the psychiatrist in close consultation with the patient, in which the GP was designated as the caregiver to be first contacted in case of warning signs.

If necessary, I can fall back on the psychiatrist directly, but often I can handle things on my own with these patients. (GP6)

If the consultant and the GP are known to each other personally, and they are aware of each other's capabilities and skills with respect to the care of the patient, a foundation for a productive collaboration develops. Some GPs say that they would like to be more involved in the continuing care of chronic psychiatric patients as long as they can fall back on secondary health care resources when there is a crisis.

## Discussion

Continuing care that is focused on the patient and his environment is the fundament of general practice. By using a systematic and structured approach, GPs provide care for patients with chronic physical diseases. Case management consists of the following elements: monitoring, assessing the physical and mental functional status of the patient, paying attention to the consequences for the family, patient (and family) education and contributing to specific, shared care with consultants.

Our study shows that the GPs refer all younger patients with a first episode psychosis, but they do not apply a proactive and structured approach when caring for patients with a chronic recurring psychosis, which is recommended in the English guideline for schizophrenia [6].

GPs experience a difference between intervening during an acute episode and caring for a chronic patient. An acute psychotic crisis is stressful and can sometimes be threatening. Often the GP feels that he cannot resolve the crisis and is dependent on the readiness of the Mental Health Service to respond. Also, GPs often find themselves in a position of solitary uncertainty when they offer to help during a psychotic crisis. The tension that these crises cause, may lead to some GPs avoiding these types of patients' altogether.

In the chronic phase, however, GPs do find satisfaction in offering patients a familiar and easy access to care. Physical complaints are often used as a means of inquiring about psychosocial circumstances, but GPs do not give systematic, structured attention to the physical and psychosocial aspects of this chronic disease. Patients with schizophrenia have a higher risk of developing somatic co-morbidities. In addition, these patients have more difficulty than other patients in obtaining access to the health services for their somatic complaints [15]. This means that the GP has to be extra vigilant regarding the appearance and existence of such signs and symptoms. Monitoring patients with schizophrenia, while paying attention to physical health and individual self-care may improve the state of health of these patients. Some GPs experience a lack of specific skills necessary to treat these patients. This finding is in agreement with the findings of other research [16].

Having sufficient professional knowledge at one's disposal does not guarantee good patient care. The physician's assets also play a role [17]. His emotions are partial determinants for how the task is performed. Anxiety, for example, can have a negative effect.

In our opinion the following conditions will be helpful for the provision of good care of psychotic patients: having compassion for this type of patient, being able to handle feelings of powerlessness, not feeling rejected too quickly, and not expecting gratitude.

An important requirement for the provision of care for these patients is a well-defined collaboration with secondary health care services. Being able to rely on a rapid response from the Mental Health Service in a crisis situation increases the GP's perception of being able to solve problems in a satisfactory manner [18]. Patient care during the chronic phase requires both parties to understand and value each other's methods and to put this knowledge to good use. GPs sometimes feel undervalued by the psychiatrists; there is a need for greater acknowledgement of the valuable and complementary role in the treatment of chronic psychoses [16]. While on the other hand, GPs have only modest interest in psychiatric diagnostic classification systems [19].

Development of GP's interest in providing care for psychotic patients could be stimulated in several ways. In the United Kingdom the National Health Service (NHS) introduced mental health indicators in the new contract with GPs as an incentive to apply a more systematic approach to care, including the use of protocols and referral guidelines [6,20].

We assume that meeting the needs GPs have, could be helpful to increase their involvement in developing a primary care guideline with specific task descriptions in the complex multidisciplinary care for psychotic patients. One of these tasks is the early detection of the first episode psychosis [20,21]. In Birmingham the Early Detection In Untreated Psychosis Trial had been designed to evaluate the effectiveness of education interventions targeted GPs on the early detection of young patients with a first episode psychosis [22].

### Study limitations and strengths

All the participating GPs regarded the meetings as positive. It enabled them to share their experience on this topic without being formally assessed. The audio tapes show that the GPs expressed themselves freely and their emotions appeared sincere. Our study included those GPs who were willing to spend time on this topic. Non-respondents were probably GPs who are not interested in care for psychotic patients. Therefore, our findings do not represent the attitudes of all GPs in The Netherlands. A quantitative sequel in a representative sample of GPs is necessary to determine the impact of these variables.

The strength of this study is the qualitative method that really captured uncensored opinions and emotions of GPs, resulting from actual experiences in this field.

## Conclusion

GPs do not apply a systematic approach to psychotic patients, but respond on requests of the patient and his family. They interpret their tasks differently, depending on personal affinities, experiences and the quality of the collaboration with the local Mental Health Service. These conclusions are the result of a qualitative study of experienced GPs and are compatible with other studies [23-25]. On the basis of these results, we propose a questionnaire for a quantitative study should be developed, which will allow the appropriate weighting factor to be allocated to the different variables. This will enable us to assess the actual needs of GPs with respect to the care for psychotic patients and to determine which interventions will contribute to further development of their competence, as well as interesting GPs in appropriating a primary care protocol.

## Competing interests

The author(s) declare that they have no competing interests.

## Authors' contributions

MO initiated and organized the study, assisted by JS. MO and JS analysed the transcripts. After reading the first draft of the manuscript all authors provided critical comments. MO and JS have written the final manuscript, which all authors read and approved.

## Pre-publication history

The pre-publication history for this paper can be accessed here:



## References

[B1] Brown S (1997). Excess mortality of schizophrenia. A meta-analysis. Br J Psychiatry.

[B2] Hennekens CH, Hennekens AR, Hollar D, Casey DE (2005). Schizophrenia and increased risks of cardiovascular disease. Am Heart J.

[B3] Burns T, Kendrick T (1997). The primary care of patients with schizophrenia: a search for good practice. Br J Gen Pract.

[B4] Citrome L, Yeomans D (2005). Do guidelines for severe mental illness promote physical health and well-being?. J Psychopharmacol.

[B5] Rowlands P (2004). The NICE schizophrenia guidelines: the challenge of implementation. Br J Psychiatry.

[B6] National Institute for Clinical Excellence (2002). Schizophrenia: full national clinical guidelines on core interventions in primary and secondary care.

[B7] WOK, Centre for Quality of Care Research (2002). De rol van de huisarts inzake geestelijke gezondheidszorg (GP's role in mental health care).

[B8] Roberts L, Roalfe A, Wilson S, Lester H (2007). Physical health care of patients with schizophrenia in primary care: a comparative study. Fam Pract.

[B9] Pope C, Mays N (1995). Reaching the parts other methods cannot reach: an introduction to qualitative methods in health and health services research. BMJ.

[B10] Kitzinger J (1995). Introducing focus groups. BMJ.

[B11] Malterud K (2001). Qualitative research: standards, challenges, and guidelines. Lancet.

[B12] Ritchie J, Spencer L, Bryman A, Burgess R (1994). Qualitative data analysis in applied policy research.

[B13] Boye B, Bentsen H, Ulstein I (2001). Relatives' distress and patients' symptoms and behaviours: a prospective study of patients with schizophrenia and their relatives. Acta Psychiatr Scand.

[B14] Tennakoon L, Fannon D, Doku V, O'Ceallaigh S, Soni W, Santamaria M, Kuipers E, Sharma T (2000). Experience of caregiving: relatives of people experiencing a first episode of psychosis. Br J Psychiatry.

[B15] Lester H, Tritter JQ, Sohoran H (2005). Patients' and health professionals' view on primary c are for people with serious mental illness: focus group study. BMJ.

[B16] Carr VJ, Lewin TJ, Barnard RE, Walton JM, Allen JL, Constable PM, Chapman JL (2004). Attitudes and roles of general practitioners in the treatment of schizophrenia compared with community mental health staff and patients. Soc Psychiatry Psychiatr Epidemiol.

[B17] Lester H, Tritter JQ, England E (2003). Satisfaction with primary care: the perspectives of people with schizophrenia. Fam Pract.

[B18] Verdoux H, Cougnard A, Grolleau S, Besson R, Delcroix F (2005). How do general practitioners manage subjects with early schizophrenia and collaborate with mental health professionals?. Soc Psychiatry Psychiatr Epidemiol.

[B19] Verhaak PFM, Schellevis FG, Nuijen J, Volers AC (2006). Patients with a psychiatric disorder in general practice: determinants of general practitioners' psychological diagnosis. Gen Hosp Psych.

[B20] Shiers D, Lester H (2004). Early intervention for first episode psychosis. BMJ.

[B21] Spencer E, Birchwood M, McGovern D (2001). Management of first-episode psychosis. Br J Psychiatry.

[B22] Tait L, Lester H, Birchwood M, Freemantle N, Wilson S (2005). Design of the BiRmingham Early Detection in UntREated psychosis Trial (REDIRECT): a cluster randomised controlled trial of general practitioner education in detection of first episode psychosis. BMC Health Services Research.

[B23] Nazareth I, King M, Davies S (1995). Care of schizophrenia in general practice: the general practitioner and the patient. Br J Gen Pract.

[B24] Lang FH, Johnstone EC, Murray DG (1997). Service provision for people with schizophrenia. Role of the general practitioner. Br J Psychiatry.

[B25] Simon AE, Lauber C, Ludewig K, Braun-Scharm H, Umbricht DS (2005). General practitioners and schizophrenia: results from a Swiss survey. Br J Psychiatry.

